# Cost-Effectiveness Analysis of Vedolizumab Compared With Infliximab in Anti-TNF-α-Naïve Patients With Moderate-to-Severe Ulcerative Colitis in China

**DOI:** 10.3389/fpubh.2021.704889

**Published:** 2021-08-20

**Authors:** Ting Zhou, Yanan Sheng, Haijing Guan, Rui Meng, Zijing Wang

**Affiliations:** ^1^School of International Pharmaceutical Business, China Pharmaceutical University, Nanjing, China; ^2^Medical Affairs, Takeda (China) International Trading Company, Beijing, China; ^3^Department of Pharmacy, Beijing Tiantan Hospital, Capital Medical University, Beijing, China; ^4^China Center for Health Economic Research, Peking University, Beijing, China

**Keywords:** ulcerative colitis, vedolizumab, infliximab, cost-effectiveness analysis, China

## Abstract

**Objective:** To evaluate the cost effectiveness of vedolizumab vs. infliximab in the treatment of anti-tumor necrosis factor-alpha (TNF-α)-naïve patients with moderate-to-severe active ulcerative colitis (UC) in China.

**Methods:** The costs and effectiveness of vedolizumab and infliximab in the treatment of anti-TNF-α naïve patients with moderate-to-severe active UC were compared using a hybrid decision tree model and a Markov model. From the perspective of the Chinese healthcare system, this study simulated the lifetime health benefits [quality-adjusted life-years (QALYs)] and costs (USD) for patients with UC from the induction phase to the maintenance phase, with an annual discount rate of 5%. The clinical efficacy and transition probability data were based on a previously published network meta-analysis. The health utility, surgical risk, biologic drug discontinuation rate, and mortality were derived from previous literature and the Chinese statistical yearbook. The cost data were based on China's drug purchase and biding platform and the results of a survey sent to clinicians in 18 tertiary hospitals. One-way and probabilistic sensitivity analyses (PSAs) were performed to validate the robustness of the models' assumptions and specific parameter estimates.

**Results:** The results of the base-case analyses showed that compared with infliximab, vedolizumab led to a gain of 0.25 QALYs (9.56 vs. 9.31 QALYs) and was less expensive by $7,349 ($180,138 vs. 187,487), indicating that the use of vedolizumab was a dominant strategy. The results of one-way sensitivity analyses suggested that the annual discount rate and health-state costs had the greatest impact, but the results were otherwise consistent with those of the base-case analyses. The PSAs suggested that vedolizumab had a 98.6% probability of being effective at a threshold of 3 times the gross domestic product (GDP) per capita in China in 2020.

**Conclusion:** Compared with infliximab, vedolizumab appears to be a more cost-effective option in the treatment of anti-TNF-α naïve adult patients with moderate-to-severe, active UC in China.

## Introduction

Ulcerative colitis (UC) is an idiopathic, chronic inflammatory bowel disease (IBD), the cause of which is attributed to the interactions between genetic and epigenetic factors, including microbial factors (1). UC is a disease of the colonic mucosa caused by an inflammatory response mediated by T-helper 2 cells, presenting with typical symptoms of blood in the stool and diarrhea (2). It's reported that approximately 6.8 million patients living with IBD in 2017 (3). The prevalence of IBD increased from 79.5 per 100,000 persons in 1990 to 84.3 per 100,000 persons in 2017 globally (3). The highest reported annual incidence and prevalence of UC in Northern Europe were 24.3 per 100,000 person-years and 505 per 100,000 person-years, respectively (2, 4). The corresponding incidence and prevalence of UC reported in Asia and the Middle East were 6.3 per 100,000 person-years and 168.3 per 100,000 person-years, respectively (4). Among Asian countries, the annual incidence of UC is relatively high in China (5), with a mean of 1.18 per 100,000 person-years in China, and the disease primarily affects male patients (sex ratio: 1.29) (5). Among Chinese UC patients, 69.8% had moderate-to-severe disease (5).

Patients with UC have been reported to have a high disease burden, a poor quality of life, increased healthcare utilization, and decreased work productivity, leading to a high economic burden (6–9). It was estimated that the mean cost of IBD was $26,255 per patient in the first year after being diagnosed in 2007–2016 in United States (10). The estimated annual economic burden of UC between 2004 to 2016 in Canada has been reported to be risen from C$6,364 to C$49,327 in the first year after being treated with anti-TNF-α drugs, furthermore, it rose to C$245,260 for UC patients in the 5 years (11). In China, the per capita medical cost of patients with IBD in 2018–2019 has been reported to be $11,668 (12).

Currently, UC disease management strategies focus on the remission of clinical symptoms and endoscopic healing in the active stages (13). Therapeutic strategies mainly include treatment with conventional drugs and biologic agents (1). Conventional therapy consists of aminosalicylic acid, corticosteroids (i.e., budesonide, prednisolone), and immunosuppressants (14), whereas biologic agents mainly include anti-tumor necrosis factor-alpha (TNF-α) drugs, such as infliximab in China. Compared with conventional therapy, biologic agents can improve clinical remission and response and mucosal healing rates during induction and maintenance treatment in patients with moderate-to-severe active UC (typically defined as a Mayo score of 6–12. Mayo score is evaluated from four categories to assess the severity of UC: stool frequency, rectal bleeding, findings of flexible proctosigmoidoscopy or colonoscopy, and physician's global assessment. Each of category is scored on a scale from 0 to 3, therefore, the maximum of total score is 12 points) (14, 15). However, 10–30% of patients with IBD have reported no response to the initial anti-TNF-α treatment, and an additional 23–46% of patients with IBD may lose their response to the treatment (16). Patients with inadequate therapeutic response may require surgical intervention, which may further increase the economic burden (17, 18).

Another biologic agent, vedolizumab, is a gut selective biologic therapy that binds exclusively to the α_4_β_7_ integrin and inhibits adhesion of lymphocytes to mucosal address in cell adhesion molecule-1 (MAdCAM-1), thereby preventing lymphocytic cells from entering the gut lamina propria and gut-associated lymphoid tissue (19). As vedolizumab exerts its effects gut selectively, the systemic anti-inflammatory effects observed with anti-TNF-α drugs are not observed after treatment with this agent (19). Previous clinical studies have reported the efficacy and adverse events of vedolizumab in anti-TNF-α-naïve and anti-TNF-α-failure patients (20, 21). In China, infliximab and vedolizumab have been approved by the Chinese National Medical Products Administration (NMPA) for the treatment of moderate-to-severe active UC. Infliximab has been used for UC for over 10 years in China, whereas vedolizumab was approved for use in March 2020. To be covered by China's national health insurance, the retail price of vedolizumab was reduced by 71% in 2020. Up to now, there's no biosimilar infliximab has been approved in China and it may have some impact on the price of infliximab in the future.

The aim of this study is to assess the cost effectiveness of vedolizumab vs. infliximab in patients with anti-TNF-α-naïve active UC from the perspective of the Chinese healthcare system and better inform healthcare decision-making in China.

## Methods

### Model Structure

**Figure 1:**
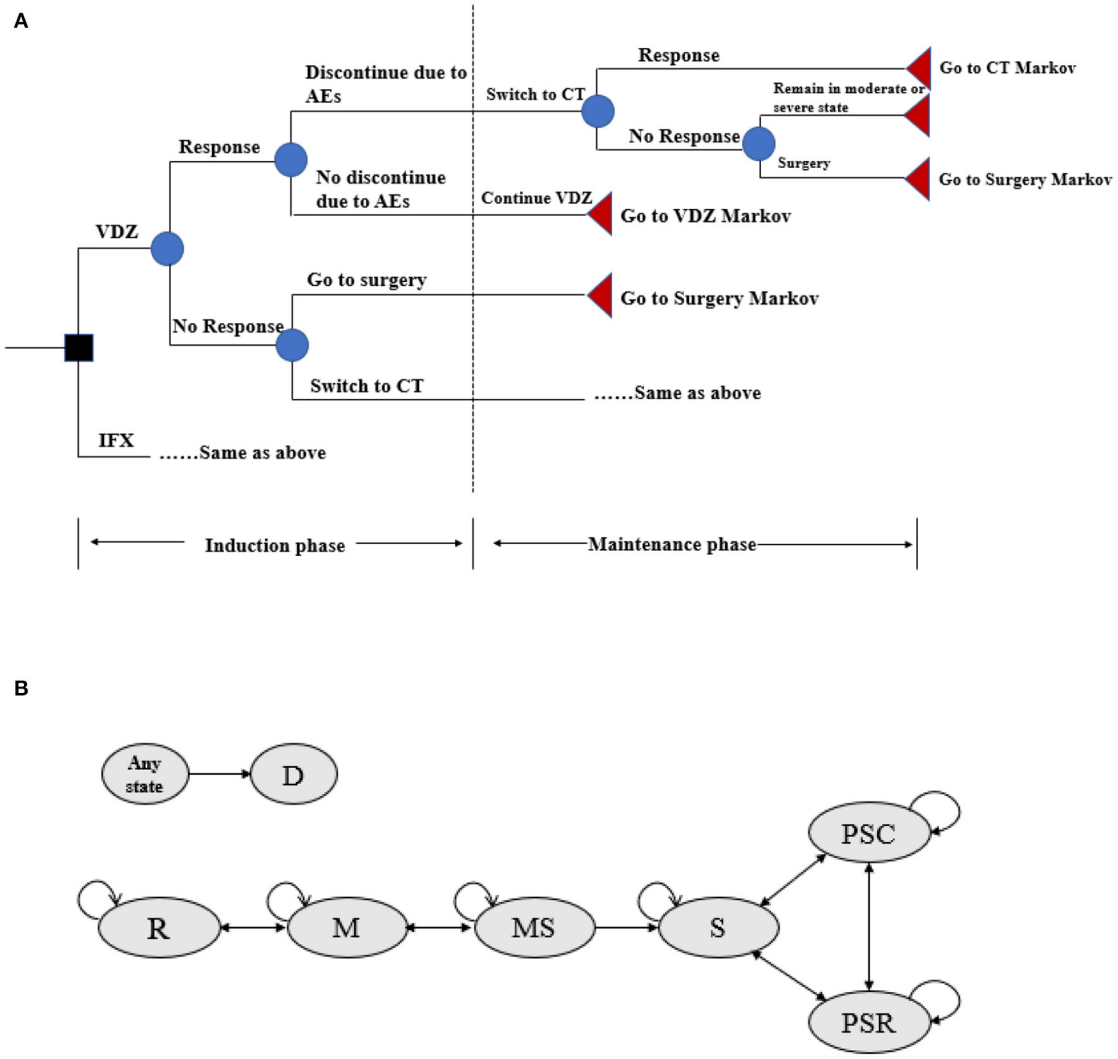
**(A)** Decision tree model structure for VDZ and IFX. AEs, adverse events; CT, conventional therapy; IFX, infliximab; VDZ, vedolizumab. Response defined as reduction in Mayo score by ≥3 or a remission Mayo score < 3. **(B)** Markov model structure of maintenance therapy. R, remission; M, mild; MS, moderate-to-severe; S, surgery; PSR, post-surgery remission; PSC, post-surgery complications; D, death.

To assess the cost effectiveness of vedolizumab vs. infliximab in patients with moderate-to-severe active UC, a hybrid simulation model was used. The model consisted of a decision tree model component for assessing the induction therapy and a Markov model component with lifetime follow-up for assessing the maintenance therapy (22). In the decision tree model, patients with UC were treated with either infliximab or vedolizumab and evaluated for treatment efficacy after a 6-week induction period. subsequently, patients who responded to the biologic without discontinuation due to intolerability of adverse events were entered into the Markov model for assessing the maintenance therapy. Conversely, patients who did not respond were switched to conventional treatment (e.g., a combination of aminosalicylic acid preparations, glucocorticoids, and immunosuppressants) within the decision tree model. Patients with a clinical response to conventional therapy then entered the Markov model for assessing the maintenance treatment with conventional drugs, whereas those who did not respond to conventional drugs persisted in the moderate-to-severe disease health state until they required surgery (Figure 1A).

For the assessment of maintenance therapy, several health states were included in the Markov model to compare the cost effectiveness of vedolizumab and infliximab (Figure 1B): remission (Mayo score: 0–2), mild (Mayo score: 3–5), moderate-to-severe (Mayo score: 6–12), surgery, post-surgery remission, post-surgery complications, and death. The cycle length was set to 8 weeks (in accordance with the administration frequency of infliximab and vedolizumab in the maintenance period in clinical trials) (20, 21), and patients could transfer from one health state to another with a certain probability that depended on the patient's health status and treatment. Death could occur at any cycle, and patients can transfer to the death state from any other state. After 1 year of treatment with vedolizumab or infliximab, patients who were still in a moderate-to-severe health state were switched to conventional treatment or surgery due to a loss of response (22). Patients who transferred to surgery would discontinue their current treatment for the remainder of their lifetime. Following surgery, these patients could subsequently experience postsurgical complications (i.e., staple line ulcers, anastomotic strictures, pouchitis), require additional surgeries, or remain in postsurgical remission (22). Patients who responded to the biologic or were in remission but discontinued the drug due to adverse event intolerability were assumed to have switched to conventional therapy during the maintenance phase.

From the perspective of the Chinese healthcare system, this model simulated the development of lifetime disease in patients with moderate to severe active UC, and the cost was reported as US dollars in 2020. Quality-adjusted life-years (QALYs) and costs were discounted at an annual rate of 5% (23).

### Model Inputs and Data Sources

#### Target Patient Population

The patient population in this study was Chinese adult patients with moderate-to-severe active UC, defined as a Mayo score of ≥6, who did not respond to conventional therapy and had not previously received anti-TNF-α therapy. Based on interviews with Chinese clinical experts (Supplementary File 1) and data from the literature (24), a hypothetical cohort of 1,000 patients (48.6% male) with an average age of 36 years old and a weight of 60 kg was simulated in the model.

#### Treatment Comparators

Among biologics, only infliximab (Remicade®, intravenous injection, 100 mg/vial, Xian Janssen) and vedolizumab (Entyvio®, intravenous injection, 300 mg/vial, Takeda) have been approved for the treatment of UC in China. Hence, vedolizumab and infliximab were included for comparison in this study. Vedolizumab was given intravenously at weeks 0, 2, and 6 of the induction period and 300 mg once every 8 weeks in the maintenance period without dose escalation. Infliximab was injected 5 mg/kg at weeks 0, 2, and 6 of the induction period and every 8 weeks in the maintenance period without dose escalation.

#### Clinical Efficacy and Transition Probability

The clinical efficacy at the end of the induction and maintenance phases was measured based on the presence of a clinical response and remission. The effect was observed at the 6th week of the induction treatment and the 52nd week of the maintenance treatment, and the proportion of patients with a clinical response or remission was evaluated. The clinical efficacy and transition probability were presented in Table 1 and Supplementary Table 1 (25, 32–34).

**Table 1:** Model inputs for clinical efficacy, adverse events, and discontinuation.

	**VDZ**	**IFX**	**CT**	**References**
**Clinical efficacy**				
At the end of induction phase				
6-week probability of response	62.35%	68.18%	34.29%	(25)
6-week probability of remission	30.25%	33.41%	8.93%	(25)
During the maintenance phase				
Annual probability of response	80.58%	56.71%	44.04%	(25)
Annual probability of remission	57.48%	31.65%	27.16%	(25)
Incidence of AEs				
Serious infection	0.48%	0.50%	3.47%	(26–29)
Tuberculosis	0.00%	0.00%	0.92%	(26–29)
Malignancy (including lymphoma)	0.04%	0.08%	0.06%	(26–29)
Acute hypersensitivity reactions	0.00%	0.00%	1.19%	(26–29)
Skin site reactions	0.29%	4.11%	4.93%	(26–29)
Probability of discontinuation				
Induction phase	3.08%	3.08%^*^	–	(27, 30, 31)
Maintenance phase per cycle	5.19%	5.19%^*^	–	(27, 30, 31)

* *Due to limited data for infliximab, similar discontinuation rates were assumed as vedolizumab*.

*VDZ, Vedolizumab; IFX, Infliximab; CT, conventional therapy*.

#### Adverse Events and Discontinuation

The types and incidence of common adverse events in different comparator regimens, and discontinuation rates were based on published literature (Table 1) (26–31). The incidence of adverse events in each cycle was assumed to remain unchanged. In addition, infliximab and vedolizumab could be withdrawn due to a lack of response or adverse event. Therefore, patients needed to stop using the biologic agent and switch to traditional therapy.

#### Mortality

In the Markov model, age- and sex-specific all-cause mortality rates were used to estimate the probability of death over time. The relationship between age and mortality was fitted by an exponential function and adjusted each cycle based on the mortality of the Chinese general population (26, 35, 36).

### Cost Estimation

In this study, the costs of biologic agents, injection management fees, costs of conventional drugs, health state costs, and costs related to the treatment of adverse events were included (Table 2). The cycle prices of vedolizumab and infliximab were based on the average price of China's provincial drug bidding for medical insurance reimbursement (37, 38). Other costs were derived from the expert interviews performed with 18 directors and deputy directors of tertiary hospitals in China (details in Supplementary File 1). The infusion management fee was $47.5 per cycle based on the clinical physician survey. For conventional treatment, the combination of different drugs and daily costs are presented in Supplementary Table 2. Among these drugs, mesalazine accounted for the highest proportion (70%), followed by prednisolone (42%), and the cost of each treatment cycle was $433.8.

**Table 2:** Model inputs for costs and health utility.

	**Costs ($)**	**Utility**	**References**
Drug cost per cycle			
Vedolizumab induction phase	2,309.1	–	(37)
Infliximab induction phase	2,791.5	–	(38)
Vedolizumab maintenance phase	769.7	–	(37)
Infliximab maintenance phase	930.5	–	(38)
Conventional therapy^*^	433.8	–	Expert Survey
Administration cost per cycle^*^			
Vedolizumab	47.5	–	Expert Survey
Infliximab	47.5	–	Expert Survey
Health-state costs per cycle^*^		Health-state utility	
Remission	129.4	0.88	(39, 40)
Mild	162.5	0.76	(39, 40)
Moderate-severe	913.2	0.42	(39, 40)
Surgery	5,905.1	0.42	(39, 40)
Postsurgery remission	1,040.0	0.60	(39, 40)
Postsurgery complications	4,673.9	0.42	(39, 40)
Costs of AEs per event^*^		Decremental AE utility	
Serious infection	1,446.2	−0.47	(41)
Tuberculosis	660.7	−0.50	(42)
Malignancy (including lymphoma)	10,389.8	−0.18	(43)
Acute hypersensitivity reactions	208.8	−0.10	(44)
Skin site reactions	39.7	−0.03	(45)

* *Cost information is based on a survey of 18 clinicians from 18 tertiary hospitals in China*.

*AE, adverse event*.

According to the Mayo score, UC can be divided into three states: remission, mild disease, and moderate-to-severe disease. Patients in the moderate-to-severe state need to be hospitalized, and those with severe UC may require surgical intervention. After surgery, patients may experience remission or complications, which could increase the treatment costs. The health state costs and costs for the treatment of a single adverse event were based on the results of the expert interviews (Supplementary File 1).

### Health Utilities

Health utility values were derived from published literature (Table 2) (39, 40). Utility decrements for adverse events were identified through a targeted review of the available published literature (41–45).

### Cost-Effectiveness Analysis

The incremental cost-effectiveness ratio (ICER) was calculated based on the lifetime costs and QALYs (calculated by multiplying the health utility and the corresponding time living with this status and discounted at each cycle) for each intervention. Half-cycle correction was used to balance the Markov model, which changed only at the end of each cycle (46). To explore the main factors affecting the results, one-way sensitivity analysis was carried out by varying one parameter at a time while all others were held constant. The variations in these parameter values were based on a credible range of estimates (95% confidence interval, or ±20% when the data were not available). For instance, a lower price (a price reduction of 20%) for infliximab was applied in the model to explore the impact on the results. A probabilistic sensitivity analysis (PSA) with 5,000 iterations was performed by varying all parameters at the same time according to pre-specified distributions. The cost parameter followed a gamma distribution, the utility and percentage parameters followed a beta distribution, and the transition probability followed a Dirichlet distribution (47). Utilizing 3 times the gross domestic product (GDP) per capita of China in 2020 ($31,500) as the upper limit of the threshold of willingness-to-pay (WTP), the economic burden of different intervention regimens was compared (23). The analysis was performed in Microsoft Excel 2019.

## Results

### Base-Case Analysis

The results of the base-case analyses of vedolizumab and infliximab are presented in Table 3. The lifetime costs associated with vedolizumab treatment were lower than those associated with infliximab treatment ($180,138 vs. 187,487; mean difference: –$7,349). Similarly, the costs for biologic therapy ($4304 vs. 5,746; mean difference: –$1,442) and of the health state ($142,326 vs. 149,182; mean difference: –$6,856) were also lower for patients treated with vedolizumab. In terms of health outcomes, vedolizumab patients were expected to gain 0.25 QALYs (9.56 QALYs vs. 9.31 QALYs) over patients treated with infliximab, which mainly contributed by more patients living with remission (2.39 QALYs vs. 1.97 QALYs) and mild (1.53 QALYs vs. 1.39 QALYs) health states. Therefore, compared with infliximab, vedolizumab appears to be a dominant strategy (more QALYs gained and less cost incurred).

**Table 3:** Base-case analysis results.

	**VDZ**	**IFX**	**Difference**
Costs	180,138	187,487	−7,349
quad Biologic therapy	4,304	5,746	−1,442
Conventional therapy	32,642	31,716	927
Health-state costs	142,326	149,182	−6,856
Adverse event costs	867	844	22
Outcomes			
QALYs	9.56	9.31	0.25
ICER ($ per QALY gained)			Dominant

*ICER, incremental cost-effectiveness ratio; VDZ, vedolizumab; IFX, infliximab; QALY, quality-adjusted life-years*.

### One-Way Sensitivity Analysis

**Figure 2:**
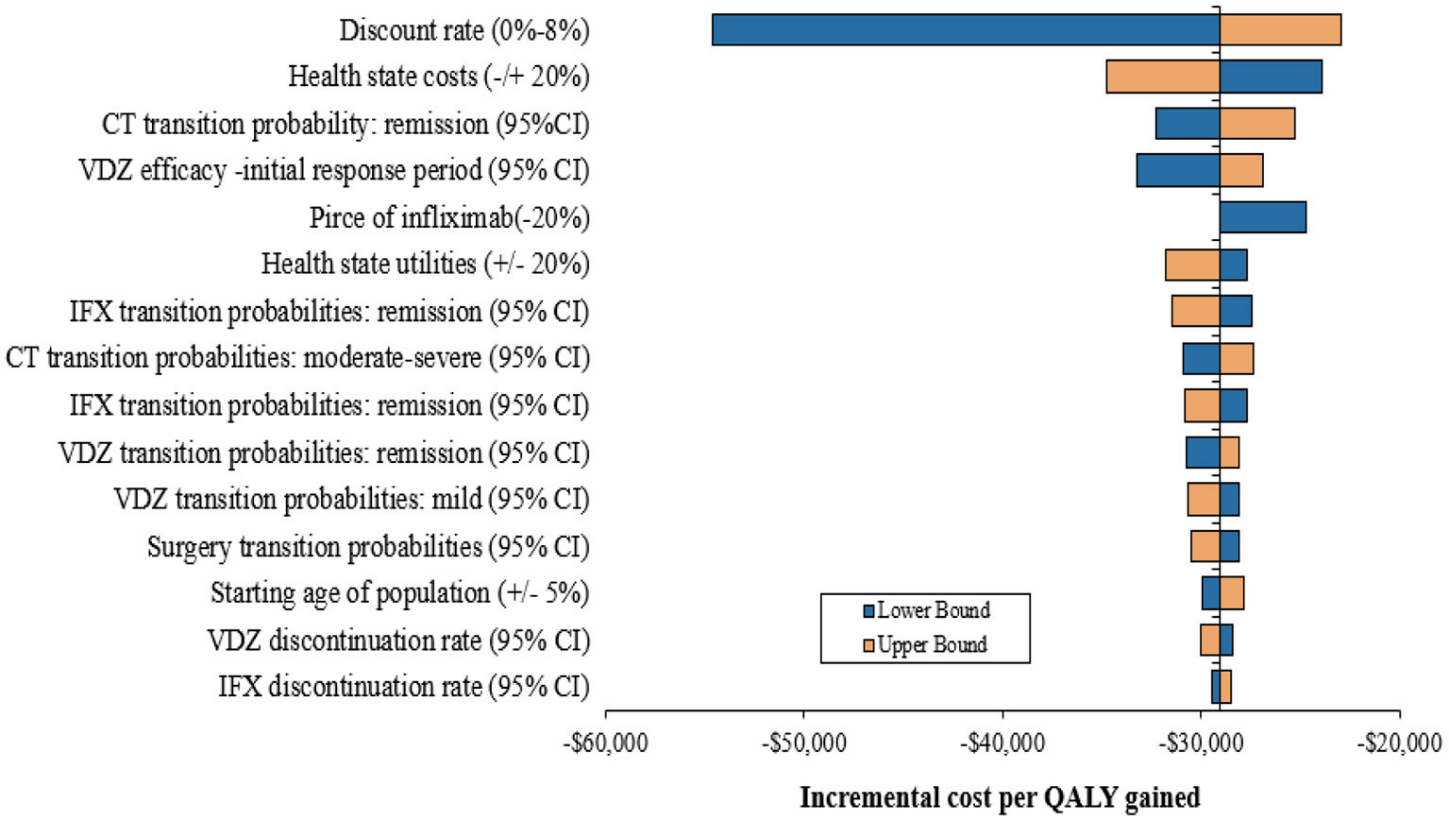
One-way sensitivity analysis results. CT, conventional therapy, IFX, infliximab, VDZ, vedolizumab; CI, confidence interval.

The results of the one-way sensitivity analyses are presented in Figure 2. The incremental cost per QALY gained was most sensitive to the change in annual discount rate, followed by health-state costs and the remission transition probability. However, the ICRE values were not over –$20,000. In addition, the reduction in the price of infliximab (20%) in the model did not have much impact on the results and the maximum value of ICER was still negative. With the variation in each factor, the results did not exceed the WTP threshold ($31,500), and compared with infliximab, vedolizumab remained cost-effective. The results of one-way sensitivity analyses suggested the robustness of the model and base-case analysis.

### Probabilistic Sensitivity Analysis

**Figure 3:**
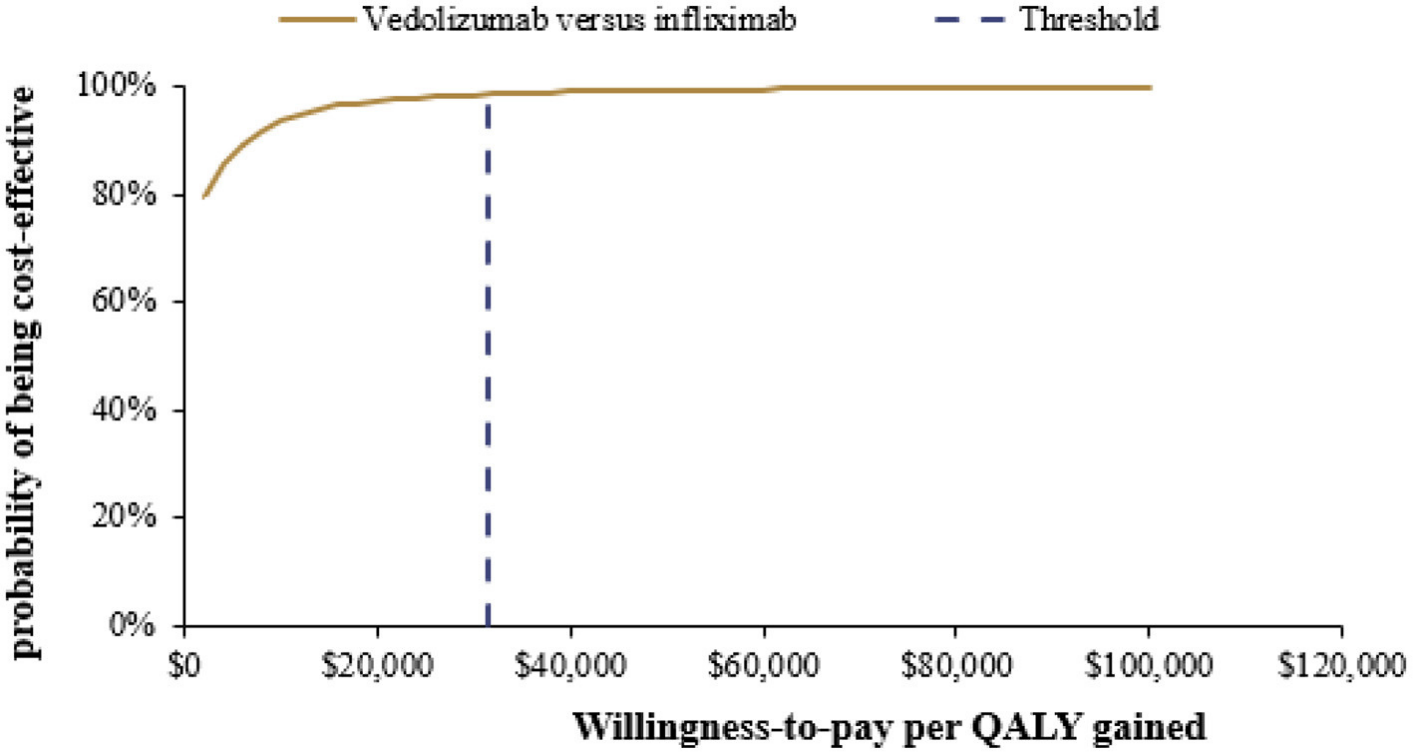
Cost-effectiveness acceptability curve for vedolizumab vs. infliximab. QALY, Quality-adjusted life-year.

After 5,000 iterations, the average lifetime costs of vedolizumab and infiximab strategies were $166,222 and 167,805 (mean difference: –$1,583), respectively. In addition, the average QALYs of vedolizumab and infiximab strategies were 9.70 QALYs and 9.40 QALYs (mean difference: 0.30 QALYs), respectively. The results of the PSA are illustrated in Figure 3. The PSA results were consistent with those of the base-case analysis, suggesting that compared with infliximab, vedolizumab had a 98.6% probability of being cost effective at a threshold of 3 times the GDP per capita in China in 2020 and that it was dominant (less costly and more effective) in 79.1% of simulations.

## Discussion

To our knowledge, this is the first study in China attempting to compare the cost effectiveness of vedolizumab and infliximab in adult patients with moderate-to-severe active UC who have had an inadequate response to, lost their response to, or were intolerant to conventional therapy using a hybrid decision tree and Markov model. The base-case analysis results suggested that, compared with infliximab, vedolizumab incurred lower costs and was expected to gain more QALYs in Chinese patients with UC. In the one-way sensitivity analysis of vedolizumab vs. infliximab, the annual discount rate was the most significant influential factor, followed by health state costs. The results of the one-way sensitivity analysis and PSA were in line with those of the base-case analysis.

The results of this study are similar to those reported in Japan (48) and the United Kingdom (UK) (25). Compared with that of infliximab, the ICER of vedolizumab over a lifetime was JP¥ 4,687,692 (about US$42,300 in 2018) per QALY gained for adult anti-TNF-α-naïve patients with moderate-to-severe active UC in Japan, and the drug was considered cost effective (48). The probability of being cost effective strategy of vedolizumab was higher compared with infliximab at a threshold of JP¥ 5,000,000 in Japan (48). The study by Wilson et al. (25) suggested that compared with infliximab, vedolizumab incurred lower costs and gained more QALYs in the UK, which is in line with the base-case analysis in our study. Furthermore, the vedolizumab was a dominant strategy in 97.6% cases compared to infliximab in the PSA at a threshold of £30,000 in the UK (25).

Under the drug review and approval policy in China, vedolizumab was approved with exemption from a domestic clinical trial by the Chinese NMPA in 2020 to accelerate access to innovative drugs. Hence, the clinical efficacy data in this model were generated from multicenter clinical trials. Since there are no head-to-head comparison studies between vedolizumab and infliximab, data from an indirect comparison network meta-analysis were used to calculate the probability of clinical response and remission (25). The response and remission probabilities for the anti-TNF-α-naïve UC population were derived from odds ratios (ORs) estimated in a mixed-treatment comparison in the meta-analysis (25). Given the clinical efficacies were not derive from direct comparison, some bias may be present in that study. However, the economic evaluation model and results were submitted to the National Institute for Health and Care Excellence (NICE) for Single Technology Appraisal in the UK, and the quality of the network meta-analysis was also assessed by NICE's Evidence Review Group (ERG) (49). In this study, the uncertainty of the data derived from that network meta-analysis was tested by performing sensitivity analysis.

Due to a lack of head-to-head comparative trials between vedolizumab and infliximab, the efficacy data of each treatment regimen were derived from a previous network meta-analysis (23). In addition, it was assumed that patients who needed surgery would stop using the biologic agent, while the probabilities of transferring to postsurgical complications, discontinuation, and death were derived from published studies (25, 32–34). The initial annual mortality rate was 0.00104 per 100,000 persons, the first cycle mortality rate was 0.00012 per 100,000 persons, and the coefficient of variation of mortality per cycle was 1.015 (35). As the disease progressed, the risk of death increased. It was assumed that the relative risks (RRs) of death in the remission and mild disease health states were the same, whereas those in the moderate-to-severe disease and surgery health states were 1.9 (36) and 1.3 (26), respectively.

For health state utility, utility weights as measured with the EQ-5D were mainly estimated based on a survey of patients with UC in Cardiff Hospital (39, 40). The study surveyed patients who had undergone surgery at least 6 weeks prior (39, 40), but the cycle length in the Markov model was 8 weeks. A survey response obtained at least 6 weeks later would not accurately reflect the quality of life for a patient who had undergone surgery during the model cycle in which the surgery occurred. Patients were expected to have a quality of life less than moderate to severe disease for the 2 weeks following the surgical procedure before progressively improving for the remainder of the cycle when they transitioned to post-surgical remission, postsurgical complications, or subsequent surgery. Therefore, patients undergoing surgery were assumed to have the same utility values as those with moderate-to-severe disease in this study.

To reduce the economic burden on patients, the coverage of high-value but high-price drugs by the national medical insurance has required negotiation with the National Healthcare Security Administration to reach an agreement after 2017 in China (50). At present, only vedolizumab and infliximab are approved for treating patients with UC and are listed in the National Reimbursement Drug List in China. Hence, these two drugs were compared in this study. To be covered by Chinese medical insurance, the retail price of vedolizumab was reduced by 71%, which mainly contributed to the cost-effective results (37). Vedolizumab and infliximab are reimbursed for anti-TNF-α Naïve patients with active UC and the co-payment varies across the provinces in China. At present, biosimilar infliximab is not available in China; the price of infliximab applied in this model was the average price of China's provincial drug bidding for medical insurance reimbursement in 2020, which is a relatively low but stable price (38). Nevertheless, the price of infliximab was reduced (20%) in the sensitivity analysis to explore the impact of price on the results, and we found that the drug remained cost effective.

This study also has some limitations. First, in the model analysis, only the direct medical cost of the treatment regimen was considered. Indirect costs and non-medical costs for the treatment of UC were not included. Therefore, the economic differences among patients receiving different treatments may have affected the results of the cost-effectiveness analysis. Second, as there are no head-to-head comparison studies for vedolizumab and infliximab, the data used in this study were derived from an indirect network meta-analysis, which may have led to bias in the economic evaluation. Third, although biologic drug discontinuation due to adverse event intolerability or loss of response was considered in this study, the data were mainly derived from randomized controlled trials, whose results may be different from those in real-world clinical practice. Fourth, due to the lack of data on the health utility value of the Chinese UC population, this study used data from published literature that reported utility values of patients in other countries. However, the sensitivity analysis results suggest that the variation in the utility values applied in the model had little impact on the cost-effectiveness analysis.

## Conclusion

This study suggests that compared with infliximab, vedolizumab appears to be a more cost-effective first-line treatment option for anti-TNF-α-naïve adult patients with moderate-to-severe active UC in China.

## Data Availability Statement

The original contributions presented in the study are included in the article/Supplementary Material, further inquiries can be directed to the corresponding author/s.

## Ethics Statement

All the data included in this analysis were derived from published literature, public data and expert survey results. No patient-identifiable data were applied or used. Therefore, institutional review board approval was not required.

## Author Contributions

TZ and HG contributed to the design of this study. TZ, YS, HG, RM, and ZW collected the data. TZ and YS performed the analysis. TZ, HG, and YS prepared the manuscript. RM and ZW helped to revise the manuscript. All authors approved the final version of this study.

## Conflict of Interest

YS is an employee of Takeda (China) International Trading Co., Ltd. The remaining authors declare that the research was conducted in the absence of any commercial or financial relationships that could be construed as a potential conflict of interest.

## Publisher's Note

All claims expressed in this article are solely those of the authors and do not necessarily represent those of their affiliated organizations, or those of the publisher, the editors and the reviewers. Any product that may be evaluated in this article, or claim that may be made by its manufacturer, is not guaranteed or endorsed by the publisher.
