# Supplementary material for: Cost-Effectiveness Analysis of Vedolizumab Compared With Infliximab in Anti-TNF-α-Naïve Patients With Moderate-to-Severe Ulcerative Colitis in China
Source: Front Public Health. 2021 Aug 20;9:704889. doi: 10.3389/fpubh.2021.704889 (PMC8417715; doi:10.3389/fpubh.2021.704889)
Supplement: Supplementary file 1 [file Table_1.DOCX]

**Supplementary Materials**

**Supplementary Table 1.** Transition probability for each treatment ^[1]^

| **Health states** | **R** | **M** | **MS** | **S** | **PSR** | **PSC** |
| --- | --- | --- | --- | --- | --- | --- |
| Vedolizumab | | | | | | |
| R | 0.93 | 0.07 | 0 | 0 | 0 | 0 |
| M | 0.243 | 0.752 | 0.005 | 0 | 0 | 0 |
| MS | 0 | 0.494 | 0.499 | 0.008 | 0 | 0 |
| S | 0 | 0 | 0 | 0.05 | 0.45 | 0.5 |
| PSR | 0 | 0 | 0 | 0.05 | 0.777 | 0.173 |
| PSC | 0 | 0 | 0 | 0.05 | 0.245 | 0.705 |
| Infliximab | | | | | | |
| R | 0.936 | 0.064 | 0 | 0 | 0 | 0 |
| M | 0.034 | 0.742 | 0.225 | 0 | 0 | 0 |
| MS | 0 | 0.221 | 0.771 | 0.008 | 0 | 0 |
| S | 0 | 0 | 0 | 0.05 | 0.45 | 0.5 |
| PSR | 0 | 0 | 0 | 0.05 | 0.777 | 0.173 |
| PSC | 0 | 0 | 0 | 0.05 | 0.245 | 0.705 |
| Conventional therapy | | | | | | |
| R | 0.936 | 0.064 | 0 | 0 | 0 | 0 |
| M | 0.062 | 0.556 | 0.382 | 0 | 0 | 0 |
| MS | 0 | 0.096 | 0.896 | 0.008 | 0 | 0 |
| S | 0 | 0 | 0 | 0.05 | 0.45 | 0.5 |
| PSR | 0 | 0 | 0 | 0.05 | 0.777 | 0.173 |
| PSC | 0 | 0 | 0 | 0.05 | 0.245 | 0.705 |
| R, remission; M, mild; MS, moderate-to-severe; S, surgery; PSR, postsurgery remission; PSC, postsurgery complications. | | | | | | |

**Reference**

[1] Wilson MR, Bergman A, Chevrou-Severac Het al. Cost-effectiveness of vedolizumab compared with infliximab, adalimumab, and golimumab in patients with ulcerative colitis in the United Kingdom. *Eur J Health Econ HEPAC Health Econ Prev Care*. 2018;19(2):229-240
