# Supplementary material for: Cost-Effectiveness Analysis of Vedolizumab Compared With Infliximab in Anti-TNF-α-Naïve Patients With Moderate-to-Severe Ulcerative Colitis in China
Source: Front Public Health. 2021 Aug 20;9:704889. doi: 10.3389/fpubh.2021.704889 (PMC8417715; doi:10.3389/fpubh.2021.704889)
Supplement: Supplementary file 2 [file Table_2.DOCX]

**Supplementary Materials**

**Supplementary Table 2.** Types, proportions and costs of conventional therapy drugs

| **CT Drugs** | **Daily cost ($)** | | **Proportion*** |
| --- | --- | --- | --- |
| Mesalazine | 7.6 | 71% | |
| Prednisolone | 1.0 | 42% | |
| Azathioprine | 2.2 | 22% | |
| Sulfasalazine | 2.9 | 11% | |
| Budesonide | 8.0 | 5% | |
| Balsalazide | 12.0 | 3% | |
| Olsalazine | 4.9 | 3% | |
| Methotrexate | 1.9 | 3% | |
| Other | 2.7 | 5% | |
| Weighted daily cost | 7.75 | | |
| cost per cycle | 433.80 | | |
| * Drugs can be combined, so the total proportion is more than 100%. The costs and proportions were derived from clinical physician surveys in 18 tertiary hospitals in China.  CT, conventional therapy. | | | |
