# Supplementary material for: Cost-Effectiveness Analysis of Vedolizumab Compared With Infliximab in Anti-TNF-α-Naïve Patients With Moderate-to-Severe Ulcerative Colitis in China
Source: Front Public Health. 2021 Aug 20;9:704889. doi: 10.3389/fpubh.2021.704889 (PMC8417715; doi:10.3389/fpubh.2021.704889)
Supplement: Supplementary file 3 [file Table_3.DOCX]

**Supplementary Materials**

**Supplementary File 1**

Number：–

**Questionnaire on the costs for the treatment of ulcerative colitis (UC) in China (Translated from the Chinese version)**

Date__________ City__________

Interviewer__________

# **General questions**

Q1. Doctor’s Name__________

Q2. Hospital_________________________

Q3. Department_______________

Q4. Hospital degree_______________

Q5. Title_______________

Q6. Experience in treating UC_______________

# **Treatment experience information**

Q7. What is the number of UC patients treated at your hospital per month？_______

Q7-1 Proportion of patients with moderate UC _______%

Q7-2 Proportion of patients with severe UC _______%

Q8. Basic information of moderate-to-severe UC patients in China

Q8-1 Average age _______years

Q8-2 Average weight_______ kg

# **Cost information**

Q9. Costs of each health state for moderate-to-severe UC patients

|  | **Health state** | **Cost per month** |
| --- | --- | --- |
| Q9-1 | Remission (Mayo score= 0-2) |  |
| Q9-2 | Mild (Mayo score = 3-5) |  |
| Q9-3 | Moderate-to-severe (Mayo score = 6-12) |  |
| Q9-4 | Surgery |  |
| Q9-5 | Postsurgery remission |  |
| Q9-6 | Postsurgery complication |  |

Q10. What are the administration costs for vedolizumab and infliximab over 8 weeks?

Q10-1 Infliximab administration cost _______

Q10-2 Vedolizumab administration cost _______

Q11**.** Adverse event costs

|  | Adverse events | Costs per event |
| --- | --- | --- |
| Q11-1 | Serious infection |  |
| Q11-2 | Tuberculosis |  |
| Q11-3 | Lymphoma |  |
| Q11-4 | Hypersensitivity |  |
| Q11-5 | Injection site reactions |  |

**Q12** Costs of conventional therapy for moderate-to-severe UC patients (including those who did not respond to biological drugs) and the proportional use of each therapeutic regimen.

|  | Treatment | Costs per month | % use |
| --- | --- | --- | --- |
| Q12-1 | Balsalazide |  |  |
| Q12-2 | Mesalazine |  |  |
| Q12-3 | Olsalazine |  |  |
| Q12-4 | Sulfasalazine |  |  |
| Q12-5 | Budesonide |  |  |
| Q12-6 | Prednisolone |  |  |
| Q12-7 | Azathioprine |  |  |
| Q12-8 | Mercaptopurine |  |  |
| Q12-9 | Methotrexate |  |  |
| Q12-10 | Other drugs |  |  |

# **Hospital list**

| Number | Hospitals included in the interview* |
| --- | --- |
| 1 | Army General Hospital |
| 2 | Bejing Jishuitan Hospital |
| 3 | Peking University People's Hospital |
| 4 | Xinhua Hospital Affiliated to Shanghai Jiao Tong University School of Medicine |
| 5 | Tongji Hospital affiliated to Tongji Medical College |
| 6 | Guangzhou First People's Hospital |
| 7 | The First Affiliated Hospital of Guang Medical University |
| 8 | Shengjing Hospital of China Medical University |
| 9 | Xijing Hospital of Air force Military Medical University |
| 10 | General Hospital of Chengdu Military Region |
| 11 | Affiliated Hospital of Chengdu University |
| 12 | Zhongnan Hospital of Wuhan University |
| 13 | Jiangsu Province Hospital |
| 14 | Shanghai General Hospital |
| 15 | Shandong Provincial Hospital |
| 16 | Bejing Shijitan Hospital |
| 17 | Hebei General Hospital |
| 18 | The First Affiliated Hospital, Sun Yat-sen University |
| *All 18 clinical physicians who were interviewed for this study worked in the respective Departments of Gastroenterology from these 18 hospitals. | |
